# Patients’ attitudes towards advance care planning (ACP) and factors affecting ACP discussion: A cross-sectional questionnaire study

**DOI:** 10.1371/journal.pone.0359632

**Published:** 2026-09-30

**Authors:** Chee Luan Lim, Jing Xin Vanessa Phua, Yi Xuan Shannon Goh, Poay Sian Sabrina Lee, Hui Ying Valerie Teo, Wern Ee Tang

**Affiliations:** 1 Medical Department, Kallang Polyclinic, National Healthcare Group Polyclinics, Singapore; 2 Clinical Research Unit, National Healthcare Group Polyclinics, Singapore; 3 Clinical Services, National Healthcare Group Polyclinics, Singapore; University of Technology Sydney, AUSTRALIA

## Abstract

**Introduction:**

The rate of advance care planning (ACP) adoption in Singapore continues to be suboptimal, despite its recognised significance. Identifying the obstacles to ACP uptake is essential for enhancing its implementation, particularly within primary care environments.

**Aim:**

To explore attitudes towards ACP and identify factors influencing ACP discussion amongst adults in public primary care setting in Singapore.

**Methods:**

A cross-sectional study was undertaken among adults aged 21 years and older at a public primary care clinic in central Singapore. Participants completed an anonymous, self-administered questionnaire available in English, Chinese, and Malay, which assessed attitudes toward ACP and factors influencing its adoption. Data were analysed using IBM SPSS Statistics version 28.0.

**Results:**

Of 471 questionnaires, 400 were returned (84.9% response rate), and 363 were fully completed. Participants’ mean age was 56 years; 58.7% were female. Ethnic distribution matched Singapore’s demographics: 72.5% Chinese, 14.0% Malay, 9.4% Indian, 4.1% others. Overall, ACP was rated as beneficial (mean score 19.96/25) and minimally burdensome (mean score 5.40/25). Fear of death was the most commonly endorsed barrier to ACP discussion (25.9%). However, fear of death was significantly less common among older adults, declining from 40.0% among participants aged 21–40 years to 14.4% among those aged ≥61 years (p < 0.001), and was also lower among participants with chronic diseases (p = 0.011) and those with family members who had experienced serious illness requiring hospitalisation (p = 0.048). Participants with history of cancer (p = 0.010) or a seriously ill family member (p = 0.001) viewed ACP more positively, while those with lower education (p = 0.023) or hypertension (p = 0.008) found ACP to be more burdensome.

**Conclusion:**

Patients in primary care generally viewed ACP positively. While fear of death was the most commonly reported barrier, it was less prevalent among older adults and those with illness-related experience. Tailored, culturally sensitive strategies may help improve ACP engagement, particularly among younger and healthier individuals.

## Introduction

Advance care planning (ACP) is described as a process in which individuals document their beliefs, values, and healthcare preferences to guide loved ones and clinicians in making decisions if they lose mental capacity [1]. Although ACP may prompt thoughts about death, its central aim is to support living well with dignity and retaining control over one’s life story until the final moments [1]. ACP can provide peace of mind for caregivers involved in planning ahead and promote family cohesion by encouraging open conversations and mutual understanding of family members’ deepest thoughts, hopes, and feelings [2].

Evidence from prior studies shows that ACP improves the quality of end-of-life care by reducing life-sustaining treatments, increasing hospice and palliative care use, and preventing unnecessary hospitalisation [3]. Recent systematic reviews and meta-reviews have further demonstrated that ACP interventions improve preparedness for future healthcare decisions and facilitate care that is more closely aligned with patients’ values and preferences [4–7]. Complex ACP interventions appear to increase adherence to patients’ documented end-of-life wishes, while a randomised controlled trial demonstrated that ACP improves end-of-life care, enhances patient and family satisfaction, and reduces stress, anxiety, and depression among surviving relatives [3,8]. Systematic reviews and observational studies further suggest that facilitated ACP may lower net healthcare costs and that extensive end-of-life discussions are associated with fewer aggressive interventions, reduced costs, and better quality of end-of-life care [9–11]. Conversely, inadequate end-of-life discussions and poor communication can lead to continued use of futile life-sustaining treatments, greater distress for patients, families, and caregivers, and a high risk that patients’ wishes are not met, as highlighted by the SUPPORT trial and other studies on discordant care [12–15]. In multicultural contexts, such as Singapore, cultural differences substantially shape attitudes and communication around end-of-life issues, underscoring the need for culturally sensitive, personalised care [16].

International data reveal that ACP adoption remains low worldwide, with large variations between countries [17]. For example, approximately one in three adults in the United States has completed an advance directive, while reported rates across Europe vary widely, from less than 1% in some populations to approximately 19% in others [18–23]. These differences may reflect variations in legislation, healthcare systems, public awareness, and cultural attitudes towards end-of-life planning. A 2021 survey across 11 high-income nations reported that only about one-quarter of older adults had completed ACP, with Germany, the United States, and Switzerland showing higher completion rates and Sweden and France among the lowest [17]. In Asia, uptake is generally lower and uneven. South Korea's relatively high uptake has been attributed in part to the implementation of the Act on Decisions on Life-Sustaining Treatment for Patients in Hospice and Palliative Care or at the End of Life in 2018, which provides a clear legal framework for advance directives and life-sustaining treatment plans, supported by a national registration system and formal procedures for documenting patients’ preferences. On the other hand, Japan, Taiwan, and Hong Kong experience more modest engagement due to limited legal standardisation, financial barriers, and low public awareness [24–27].

The low ACP uptake in many Asian settings is attributed to interacting cultural, systemic, and individual barriers [25]. Culturally, taboos around discussing death, a strong emphasis on family-centred decision-making, and prioritising family harmony over explicit expression of individual preferences hinder open ACP conversations [25,28,29]. System-level obstacles include limited public awareness, inconsistent or absent legal frameworks for advance directives, and inadequate training of healthcare professionals to conduct end-of-life discussions [25,30]. On a personal level, psychological resistance to confronting mortality, poor understanding of ACP benefits, and the perception that ACP is only relevant in advanced illness further dampen engagement [25].

Against this backdrop, Singapore faces rapid population ageing, with one in four residents expected to be aged 65 years or older by 2030, leading to more complex care needs and prolonged periods of disability [31]. ACP in Singapore is a national programme for adults aged 21 and above, delivered through multiple community touchpoints such as community centres, polyclinics, and hospitals, yet actual completion remains low despite its potential to improve end-of-life care [32,33]. Earlier data showed only 5,100 ACPs completed between 2011 and 2015 and just 7 of 406 community-dwelling respondents reporting an ACP discussion in a 2017 survey, while more recent figures indicate 26,500 completed ACPs over five years and over 50,000 by October 2024 following a multi-agency campaign, though uptake is still modest compared with other countries [33–36].

Few ACP-related surveys have been conducted in local primary care, and this study is presented as the first in Singapore to examine patients’ attitudes towards ACP and factors influencing ACP discussions specifically in a primary care setting, aiming to generate insights to guide better implementation strategies.

## Methodology

### Study design and setting

This was a cross-sectional, anonymous, self-administered survey conducted among participants attending Kallang Polyclinic, one of the nine National Healthcare Group Polyclinics (NHGP) in Singapore. The National Healthcare Group (NHG) is one of the three integrated healthcare clusters in Singapore. NHG Polyclinics serves as the primary care arm, providing comprehensive primary care services in the central and northern regions of Singapore [37]. Recruitment commenced on 22 November 2024 and ended on 3 February 2025.

### Participants

Eligible adults aged 21 and above, who were able to understand English, Mandarin, or Malay were invited to participate in the study. To ensure data completeness and comprehension, participants were offered the questionnaire in their preferred language (English, Mandarin, or Malay).

Participants were recruited through (1) referral by healthcare providers through convenience sampling or (2) direct approach by research team members in the clinic waiting area. Those who agreed to participate completed an anonymous, self-administered questionnaire which took approximately 10–15 minutes to complete before depositing into a secured collection box accessible by authorised study team members. Participation in the research study was voluntary. Participants could withdraw at any time by choosing not to complete the questionnaire or skipping any questions they were not comfortable answering.

### Ethics approval

This study was reviewed and approved by the Domain Specific Review Board (DSRB) of National Healthcare Group (NHG), Singapore (DSRB Reference: 2024–4313). Since it is an anonymous questionnaire which does not collect any identifiable information, implied consent was sufficient from all recruited participants for this study.

### The measurement tool

The questionnaire consisted of four sections: A, B, C and D.

- Section A comprised of study information which included study subject number and date of survey.
- Section B comprised of demographics and participants’ experiences with illness (either personal or family members), Demographic data obtained included age, sex, ethnicity, religion, current marital status, education level, work status, housing type, residential status, whether the participants had children, persons that participants live with and presence of chronic diseases.
- Section C and D were adapted from Cheng *et al*. [38] to assess attitudes towards ACP and factors affecting ACP discussion respectively. Cheng et al. developed their questionnaire based on the Theory of Reasoned Action, incorporating belief and evaluation constructs to measure attitudes towards ACP. Questionnaire items were generated from literature review and expert input, followed by content validation and psychometric testing to establish reliability and validity before implementation. Permission to the questionnaire was obtained from the original authors prior to study commencement.
- The questionnaire was also translated into Chinese and Malay languages using a forward and backward translation approach. A copy of the questionnaire is included in the Appendix section for reference.

Section C measured participants’ attitudes towards ACP through 12 statements; each assessing belief (Bi) and evaluation (Ei) components. Both the Bi and Ei were rated using a 5-point Likert scale: Bi was measured using a scale ranging from “strongly disagree” (1 point) to “strongly agree” (5 points), Ei was measured using a scale ranging from “very unimportant” (1 point) to “very important” (5 points). The overall Attitude score was calculated using a formula Attitude = Bi x Ei, with a higher Attitude score indicating more a positive attitude of the participant towards ACP and vice versa. Among the 12 statements, statement 1, 2, 3, 4, 5, 7, and 8 assessed “benefits of ACP” whereas statement 6, 9, 10,11 and 12 assessed “burdens of ACP”.

Section D comprised of eight statements exploring factors that may affect ACP discussion. Responses to these statements were scored using a 5-point Likert scale, with scores ranging from “strongly disagree” (1 point) to “strongly agree” (5 points).

Test-retest reliability was assessed by ten nonmedical individuals who were not part of the study team, over a two-week interval. Intraclass Correlation Coefficient (ICC) was found to be 0.877 (95% CI: 0.737–0.963) for section C (attitudes towards ACP) and 0.713 (95% CI: 0.367–0.915) for section D (factors affecting ACP discussion). Internal consistency was assessed using full study sample (n = 363), with the Cronbach’s alpha of 0.916 for benefits of ACP (seven items) and 0.869 for burdens of ACP (five items). The Cronbach’s alpha was 0.800 for attitudes towards ACP and 0.872 for factors affecting ACP discussion. A panel consisting of one Palliative Medicine specialist and two Consultant Family Physicians, each with experience in end-of-life discussions, were consulted to ensure content and face validity of the questionnaire.

### Sample size calculation

Sample size was calculated based on 20:1 participant-to-variable ratio involving multivariable regression with 17 variables, resulting in a target of 320 participants. To account for an anticipated 15% missing data rate, we increased the sample size to 391 and ultimately rounded it to 400 to ensure robustness and reliability.

### Statistical analysis

Descriptive statistics including mean, standard deviation (SD), median, and interquartile range (IQR) were used to characterise the variables in the questionnaire. Tests of normality indicated that the data were not normally distributed. Therefore, medians were reported in Table 2 to appropriately represent the non-normal distribution of the data. However, means and standard deviations were presented in Tables 3 and 5 to facilitate comparison with the study by Cheng *et al*. Variance Inflation Factor (VIF) was calculated to assess multicollinearity among predictors, with all values generally below 3, indicating acceptable levels of collinearity. Non-parametric tests were employed to analyse between-group differences. Specifically, the Kruskal-Wallis test was used to assess differences across multiple independent groups, while the Dunn’s post hoc with Bonferroni correction was applied following a significant result from the Kruskal Wallis-test for pairwise comparisons between independent groups**.** The collected data were further subjected to regression analysis to examine relationships between variables. Multivariable linear regression analyses were performed to identify factors independently associated with ACP benefit and burden scores. Regression coefficients (β), 95% confidence intervals (CI), and p-values were reported. Statistical significance for all analyses was established at p < 0.05. Data analysis was conducted using IBM SPSS Statistics 28.0.

## Results

A total of 487 individuals were approached, 16 individuals were excluded as they were under the age of 21, and 71 declined to participate, citing reasons such as “rushing for time”, “not keen to participate”, and “afraid of missing queue number”. 400 individuals agreed to participate in the study (85% response rate). Of these, 37 returned incomplete questionnaires and were excluded from the analysis. As a result, a total of 363 completed surveys were included in the final analysis. The recruitment process is shown in the study flow chart in Fig 1.

**Fig 1:**
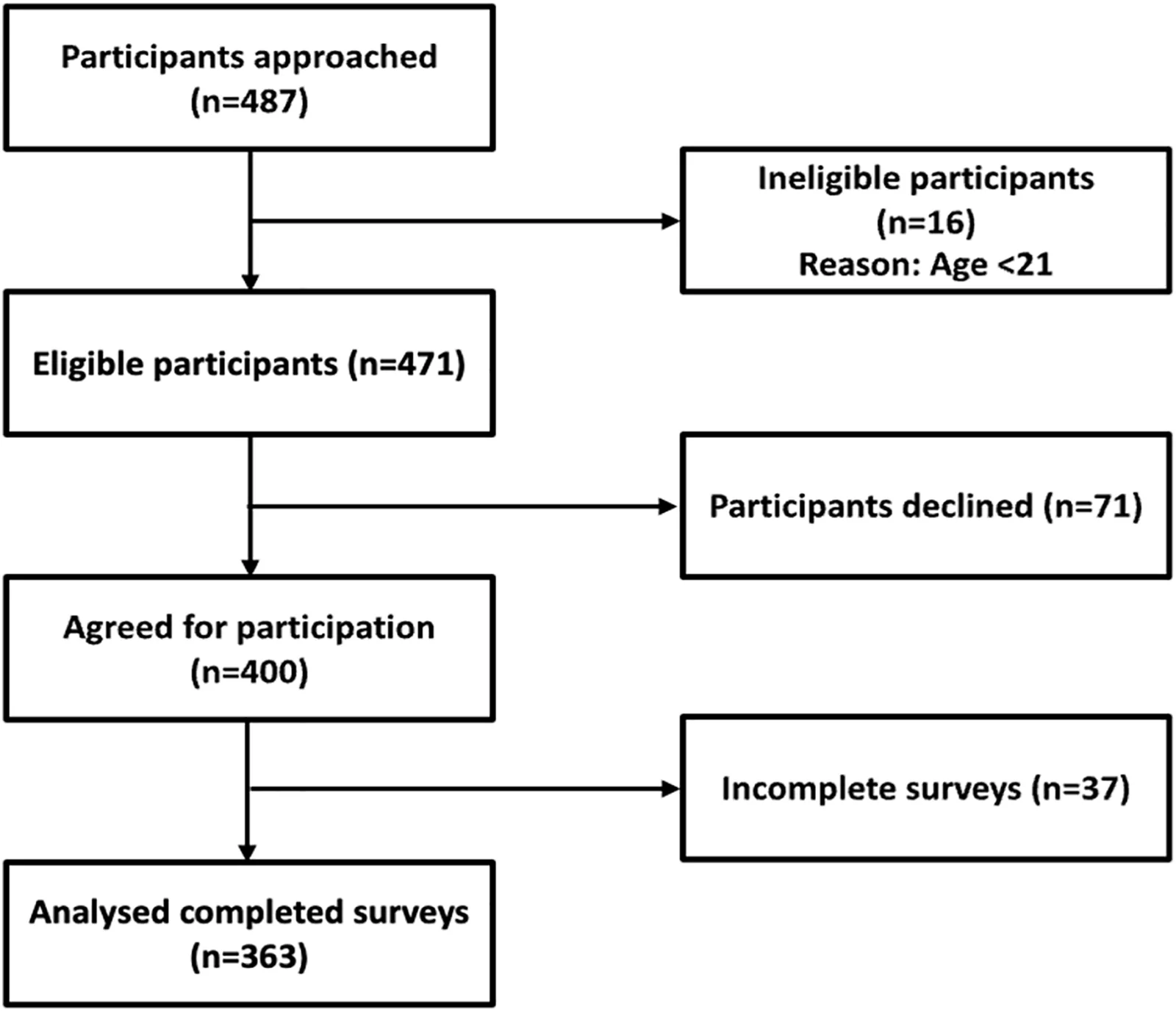
Participant recruitment and study inclusion flow diagram.

The demographic profile of the 363 participants showed an even age distribution where approximately one third were aged between 21–40 years old (33.1%), another third were 41–60 years old (32.5%), and the remaining third were 61 years old and above (34.4%). 41.3% of participants were males and 58.7% females. Most participants were of Chinese ethnicity (72.5%), followed by Malay (14.0%), Indian (9.4%), and other ethnicities (4.1%), reflecting the Singapore ethnic composition. Most participants were currently married (60.6%), while 27.3% had never married, and 12.1% were separated, divorced, or widowed. Majority of participants had attained a university education (40.5%), were employed (67.2%), and live in HDB 4/5-room flats, executive flats, or studio apartments (50.1%). A significant portion had children (63.1%) and did not live alone (91.7%). Most participants had not experienced a serious illness requiring hospitalisation in the past (82.1%), However, over half had family members with a history of serious illness requiring hospitalization (53.4%). 191 (52.6%) of the participants reported having at least one chronic medical condition. The demographic characteristics of participants were summarised in Table 1.

**Table 1:** Demographic characteristics (N = 363).

Demographics	n (%)	Demographics	n (%)
**Age**		**Dwelling**	
21-40	120 (33.1)	HDB 1/2/3-room flat	91 (25.1)
41-60	118 (32.5)	HDB 4/5-room flat / Executive Flat / Studio Apartment	182 (50.1)
61 and above	125 (34.4)	Landed / Condominiums	
		Others	84 (23.1)
			6 (1.7)
**Gender**		**Children**	
Female	213 (58.7)	Yes	229 (63.1)
Male	150 (41.3)	No	134 (36.9)
**Ethnicity**		**Living with**	
Chinese	263 (72.5)	Living alone	30 (8.3)
Malay	51 (14)	Not living alone	333 (91.7)
Indian	34 (9.4)		
Others	15 (4.1)		
**Religion**		**Chronic conditions**	
With religion	302 (83.2)	Yes	172 (47.4)
Without religion	61 (16.8)	No	191 (52.6)
**Marital status**		**Chronic conditions**	
Married	220 (60.6)	Cancers	9 (2.5)
Never married Divorced/Separated/Widowed	99 (27.3)	Stroke	12 (3.3)
	44 (12.1)	Diabetes mellitus	64 (17.6)
		Hypertension	102 (28.1)
		Hyperlipidaemia	104 (28.7)
**Highest educational level**		**Personal serious illness requiring hospitalisation**	
No formal education / Primary	39 (10.7)	Yes	
Secondary / ‘N’ level / ‘O’ level	73 (20.1)	No	65 (17.9)
‘A’ level / Diploma	103 (28.4)		298 (82.1)
University and above	147 (40.5)		
Others	1 (0.3)		
**Work status**		**Family members with serious illness requiring hospitalisation**	
Employed	244 (67.2)	Yes	
Unemployed	119 (32.8)	No	169 (46.6)
			194 (53.4)

Table 2 summarises how perceived benefits and burdens of ACP vary across demographic groups. Statistically significant differences in perceived benefits were observed across several categories: age (higher among individuals aged 41–60 and ≥61 years, p = 0.005), marital status (higher in married participants, p = 0.034), parental status (increased among those with children, p = 0.042), chronic conditions (notably higher in cancer patients, p = 0.005, and hypertensive individuals, p = 0.039), and family medical history (greater in respondents with family members hospitalized for serious illness, p = 0.002). Post-hoc pairwise comparisons using Dunn’s test with Bonferroni correction was conducted for age and marital status.

**Table 2:** Overall scores of perceived benefits and burdens towards ACP.

Demographics	Benefits		Burdens	
	Median (IQR)	p-value	Median (IQR)	p-value
**Age**		**0.005**		0.73
21-40	19.93 (8.36)		4.20 (4.55)	
41-60	23.57 (6.57)		4.30 (4.50)	
61 and above	22.43 (9.00)		4.20 (5.50)	
**Gender**		0.83		0.31
Female	21.86 (8.21)		4.20 (4.30)	
Male	22.14 (9.00)		4.20 (5.50)	
**Ethnicity**		0.52		0.61
Chinese	22.00 (8.43)		4.20 (4.40)	
Malay	20.57 (9.71)		4.20 (6.80)	
Indian	22.57 (8.79)		3.20 (6.00)	
Others	19.42 (9.43)		5.00 (5.40)	
**Religion**		0.75		0.85
With religion	21.86 (9.00)		4.20 (4.90)	
Without religion	22.29 (7.71)		4.00 (4.00)	
**Marital status**		**0.034**		0.43
Married	22.43 (8.25)		4.20 (5.20)	
Never married	20.29 (8.29)		4.20 (4.20)	
Divorced/Separated/Widowed	19.43 (11.18)		4.80 (5.15)	
**Highest educational level**		0.21		0.07
No formal education / Primary	22.43 (9.71)		4.20 (5.60)	
Secondary / ‘N’ level / ‘O’ level	20.71 (10.50)		5.00 (6.50)	
‘A’ level / Diploma	22.43 (8.29)		4.60 (5.00)	
University and above	21.86 (7.86)		4.00 (4.20)	
Others				
**Work status**		0.81		0.92
Employed	21.71 (8.25)		4.20 (4.70)	
Unemployed	22.14 (9.00)		4.20 (4.40)	
**Dwelling**		0.51		0.68
HDB 1/2/3-room flat	21.29 (9.00)		4.20 (4.80)	
HDB 4/5-room flat / Executive Flat / Studio Apartment	21.50 (9.00)		4.20 (5.05)	
Landed / Condominiums				
Others	22.29 (6.54)		4.60 (4.40)	
	23.93 (10.18)		4.30 (3.15)	
**Children**		**0.042**		0.96
Yes	22.43 (8.43)		4.20 (4.90)	
No	20.36 (9.00)		4.20 (4.45)	
**Living with**		0.73		0.72
Living alone	22.29 (10.32)		4.10 (3.55)	
Not living alone	21.86 (8.79)		4.20 (4.80)	
**Chronic conditions**				0.383
Yes	22.71 (9.00)	0.15	4.40 (5.35)	
No	20.43 (8.71)		4.20 (4.60)	
**Chronic conditions**				
Cancers	25.00 (1.29)	**0.005**	3.00 (3.60)	0.19
Stroke	23.64 (9.00)	0.58	1.30 (6.45)	0.08
Diabetes mellitus	22.71 (8.71)	0.43	4.00 (4.80)	0.18
Hypertension	22.86 (7.71)	**0.039**	4.40 (6.40)	0.16
Hyperlipidaemia	23.29 (8.79)	0.1	4.20 (5.40)	0.81
**Personal serious illness requiring hospitalisation**		0.97		0.85
Yes				
No	21.29 (8.50)		4.20 (4.80)	
	22.07 (9.00)		4.20 (4.50)	
**Family members with serious illness requiring hospitalisation**		**0.002**		0.81
Yes				
No	22.71 (7.71)		4.20 (4.80)	
	20.86 (9.61)		3.20 (4.60)	

### Age

Significant differences in perceived benefits were found across age groups (p = 0.005) with middle-aged adults (41–60 years) reporting the highest median benefit score [median (IQR): 23.57 (6.57), out of 25], followed by older adults (≥61 years) [median (IQR): 22.43 (9.00), out of 25], and young adults (21–40 years) with the lowest [median (IQR): 19.93 (8.36). Pairwise comparisons using Dunn’s test with Bonferroni correction showed that the difference between young and middle-aged adults was statistically significant (adjusted p = 0.003), while differences between young and older adults (adjusted p = 0.589) and between middle-aged and older adults (adjusted p = 0.135) were not. This suggests that middle-aged adults perceive significantly greater benefits from ACP compared to young adults.

### Marital status

While initial analysis revealed statistically significant differences in perceived ACP benefits across different marital status groups (p = 0.034), with married individuals reporting the highest benefit score [median (IQR): 22.43 (8.25), out of 25] compared to never married [median (IQR): 20.29 (8.29), out of 25] (20.29, IQR = 8.29) and divorced/separated/widowed individuals [median (IQR): 19.43 (11.18), out of 25), subsequent pairwise comparisons with adjusted p-values showed no statistically significant differences between specific groups. The adjusted p-values for comparisons between married and never married (p = 0.095), married and divorced/separated/widowed (p = 0.17), and never married and divorced/separated/widowed (p > 1.00) all exceeded the significance threshold, suggesting that while married individuals tend to perceive greater benefits from ACP, these differences were not statistically significant when corrected for multiple testing.

### Other demographic factors

Perceived benefits were significantly higher among participants with children (p = 0.042), individuals with cancer (p = 0.005) or hypertension (p = 0.039), and those with a family history of serious illness requiring hospitalisation (p = 0.002). These results indicate that personal and familial health experiences may heighten the appreciation for the benefits of ACP. It was interesting to note that while several demographic variables were significantly associated with perceived benefits of ACP, none were significantly associated with perceived burdens (all p-values >0.05). This suggests that while certain life experiences and health conditions may enhance the perceived benefits of ACP, these did not appear to influence the perceptions of its burdens.

Table 3 presents participants’ attitudes towards the benefits of ACP ranked from highest to lowest mean scores. The most highly rated benefit was *informing patients about their condition for future preparation* [mean 21.67 (SD 5.83)]. Other highly rated perceived benefits were *reducing the burden of care on family or friends* [mean 20.55 (SD 6.27)], *informing family or friends of one’s wishes* and *encouraging them to follow them* [mean 20.29 (SD6.37)], *reducing unnecessary pain and suffering at the end of life* [mean 19.87 (SD 6.69)], *helping maintain quality of life* [mean 19.30 (SD 6.91)], *reducing conflict when family or friends make medical decisions* [mean 19.21 (SD 6.79)], and *reducing the burden of future medical decision-making for family or friends* [mean 18.84 (SD 6.72)]. The overall mean score for perceived benefits was 19.96 (SD 5.46), reflecting generally positive attitudes towards ACP among participants.

**Table 3:** Attitudes towards ACP – BENEFITS.

Ranking	Benefits of ACP	Mean (SD)
**1**	I believe that patients should be adequately informed about their condition so that they can prepare for the future.	21.67 (5.83)
**2**	I believe that discussing advance care planning can reduce the burden of care on my family or friends.	20.55 (6.27)
**3**	I believe that discussing advance care planning will inform my family or friends of my wishes and encourage them to follow them.	20.29 (6.37)
**4**	I believe that discussing advance care planning with my family or friends will reduce unnecessary pain and suffering at the end of my life.	19.87 (6.69)
**5**	I believe that discussing advance care planning with my family or friends will help maintain my quality of life.	19.30 (6.91)
**6**	I believe that discussing advance care planning with my family or friends will reduce conflict when they make medical decisions on my behalf in the future.	19.21 (6.79)
**7**	I believe that discussing advance care planning with family or friends will reduce the burden of making medical decisions for me in the future.	18.84 (6.72)
**Total**		**19.96 (5.46)** Out of 25

Table 4 presents participants’ attitudes towards the burdens of ACP ranked from highest to lowest. The most endorsed perceived burden was *discussing ACP with family or friends will make them uncomfortable* [mean 8.46 (SD 6.61)]. Other concerns include the belief that *ACP discussions will upset the participant* [mean 5.89 (SD 6.04)], *upset their doctor* [mean 4.93 (SD 5.30)], *make them feel hopeless* [mean 4.19 (SD 5.36)], or *bring bad luck* [mean 3.52 (4.56)]. The overall mean score for perceived burdens was 5.4 (SD 4.39), suggesting that generally participants perceived the burdens of ACP as low.

**Table 4:** Attitudes towards ACP – BURDENS.

Ranking	Burdens of ACP	Mean (SD)
**1**	I believe that discussing advance care planning with my family or friends will make them uncomfortable.	8.46 (6.61)
**2**	I believe that discussing advance care planning will upset me.	5.89 (6.04)
**3**	I believe that discussing advance care planning will upset my doctor.	4.93 (5.3)
**4**	I believe that discussing advance care planning will make me feel hopeless.	4.19 (5.36)
**5**	I believe that discussing advance care planning will bring me bad luck.	3.52 (4.56)
**Total**		**5.4 (4.39)** Out of 25

Table 5 presents factors influencing ACP discussion among 363 participants, ranked by mean score on a 5-point Likert scale. Although fear of death was the highest-ranked factor among those surveyed, its overall endorsement was low, with a mean score of 1.83 (SD 0.81). Only 25.9% of participants agreed or strongly agreed that they were terrified of death, while 31.4% were neutral and 42.7% disagreed or strongly disagreed. Taken together, 74.1% of respondents did not actively endorse fear of death as a barrier to ACP discussion. Likewise, all remaining factors demonstrated low mean scores (1.29–1.47), with agreement rates below 15%. These findings suggest that none of the surveyed factors constituted a major barrier to ACP discussions for most participants, although fear of death was relatively more frequently endorsed than the other factors examined. These factors included beliefs that ACP is unnecessary because: *one will make their own medical decisions* [mean 1.47 (SD 0.74), 14.9% agree], *family will make the best decisions* [mean 1.44 (SD 0.69), 11.3% agree], *doctors won't perform unnecessary procedures* [mean 1.42 (SD 0.69), 11.6% agree], *ACP is only for older or sicker people* [mean 1.37 (SD 0.67), 10.5% agree], *doctors already know patients’ wishes* [mean 1.35 (SD 0.64), 9.1% agree], *suffering has purpose* [mean 1.32 (SD 0.62), 8.0% agree], and *ACP disrupts divine arrangements* [mean 1.29 (SD 0.62), 8.5% agree]. For each of these statements, a majority of participants (67.8%−79.3%) disagreed, suggesting that most respondents do not view these beliefs as meaningful barriers to ACP discussion.

**Table 5:** Factors affecting ACP discussion (N = 363).

Ranking	Item	Mean (SD) Out of 5	Frequency, n (%)		
			Agree to Strongly Agree	Neutral	Disagree and Strongly Disagree
1	I am terrified of death.	1.83 (0.81)	94 (25.9)	114 (31.4)	155 (42.7)
2	I believe that signing advance care planning is unnecessary because I will make my own medical decisions when needed.	1.47 (0.74)	54 (14.9)	62 (17.1)	247 (68.0)
3	I believe that signing advance care planning is unnecessary because my family will make the best decisions for me.	1.44 (0.69)	41 (11.3)	76 (20.9)	246 (67.8)
4	I believe that signing advance care planning is unnecessary because the doctor will not perform unnecessary medical procedures when I am old, such as intubation with a ventilator.	1.42 (0.69)	42 (11.6)	68 (18.7)	253 (69.7)
5	I believe that signing advance care planning is only necessary for people older or sicker than me.	1.37 (0.67)	38 (10.5)	57 (15.7)	268 (73.8)
6	I believe that signing advance care planning is unnecessary because my doctor knows what I want in terms of my future care.	1.35 (0.64)	33 (9.1)	62 (17.1)	268 (73.8)
7	I believe that I do not need to sign advance care planning because all suffering has a purpose.	1.32 (0.62)	29 (8.0)	59 (16.3)	275 (75.8)
8	I believe that signing advance care planning will disrupt divine arrangements that God (or fate or any particular deity) has for me.	1.29 (0.62)	31 (8.5)	44 (12.1)	288 (79.3)

To further explore the most frequently endorsed factor affecting ACP discussion, associations between demographic characteristics and the statement “I am terrified of death” were examined (Table 6). Fear of death was significantly associated with age group (p < 0.001), marital status (p = 0.025), employment status (p = 0.018), living alone (p < 0.001), chronic disease status (p = 0.011), and having a family member with a serious illness requiring hospitalisation (p = 0.048). A clear age gradient was observed, with agreement that one was terrified of death decreasing from 40.0% among participants aged 21–40 years to 23.7% among those aged 41–60 years and 14.4% among those aged 61 years and above. Participants with chronic diseases and those with family members who had experienced serious illness were also less likely to report fear of death. No significant associations were observed for gender, ethnicity, religion, education level, having children, or personal history of serious illness requiring hospitalisation.

**Table 6:** Association between fear of death and demographic characteristics (N = 363).

	Frequency, n (%)			
Variable	Strongly Agree and Agree	Neutral	Disagree and Strongly Disagree	p-value
**Age group**				<0.001
21–40 years (n = 120)	48 (40.0)	40 (33.3)	32 (26.7)	
41–60 years (n = 118)	28 (23.7)	39 (33.1)	51 (43.2)	
≥61 years (n = 125)	18 (14.4)	35 (28.0)	72 (57.6)	
**Gender**				0.152
Male (n = 150)	31 (20.7)	49 (32.7)	70 (46.7)	
Female (n = 213)	63 (29.6)	65 (30.5)	85 (39.9)	
**Ethnicity**				0.689
Chinese (n = 263)	67 (25.5)	86 (33.0)	110 (41.8)	
Non-Chinese (n = 100)	27 (27.0)	28 (28.0)	45 (45.0)	
**Religion**				0.127
With religion (n = 302)	76 (25.2)	90 (29.8)	136 (45.0)	
Without religion (n = 61)	18 (29.5)	24 (39.3)	19 (31.1)	
**Marital status**				0.025
Married (n = 220)	61 (27.7)	72 (32.7)	87 (39.5)	
Not married (n = 99)	27 (27.3)	33 (33.3)	39 (39.4)	
Divorced/Separated/Widowed (n = 44)	6 (13.6)	9 (20.5)	29 (65.9)	
**Education**				0.675
University and above (n = 147)	41 (27.9)	47 (32.0)	59 (40.1)	
University below and others (n = 216)	53 (24.5)	67 (31.0)	96 (44.4)	
**Employment status**				0.018
Employed (n = 244)	71 (29.1)	81 (33.2)	92 (37.7)	
Unemployed (n = 119)	23 (19.3)	33 (27.7)	63 (52.9)	
**Children**				0.124
Yes (n = 229)	56 (24.5)	66 (28.8)	107 (46.7)	
No (n = 134)	38 (28.4)	48 (35.8)	48 (35.8)	
**Living alone**				<0.001
Yes (n = 30)	1 (3.3)	6 (20.0)	23 (76.7)	
No (n = 333)	93 (27.9)	108 (32.4)	132 (39.6)	
**Chronic diseases**				0.011
Yes (n = 172)	34 (19.8)	52 (30.2)	86 (50.0)	
No (n = 191)	60 (31.4)	62 (32.5)	69 (36.1)	
**Personal serious illness requiring hospitalisation**				0.151
Yes (n = 65)	11 (16.9)	25 (38.5)	29 (44.6)	
No (n = 298)	83 (27.9)	89 (29.9)	126 (42.3)	
**Family members with serious illness requiring hospitalisation**				0.048
Yes (n = 169)	35 (20.7)	62 (36.7)	72 (42.6)	
No (n = 194)	59 (30.4)	52 (26.8)	83 (42.8)	

Table 7 presents the associations between demographics and health-related factors and attitudes towards the benefits of ACP. Overall, the analysis showed that most sociodemographic and health-related factors were not significantly associated with attitudes towards the benefits of ACP. Age, gender, ethnicity, religion, marital status, education level, family structure, living arrangements, and common chronic conditions such as diabetes, hypertension, hyperlipidaemia, and stroke did not demonstrate significant associations. In contrast, respondents with a history of cancer (p = 0.010) had significantly more positive attitudes towards the benefits of ACP, as did those who had family members with a serious illness requiring hospitalisation (p = 0.001).

**Table 7:** Multivariable linear regression of factors associated with perceived ACP benefit score.

Demographics	B (95% CI)	p-value
**Age**		
21-40	Reference	-
41-60	1.31 (-2.19, 1.41)	0.671
61 and above		0.089
	-0.39 (-0.20, 2.83)	
**Gender**		
Male	Reference	-
Female	0.70 (-0.46, 1.86)	0.236
**Ethnicity**		
Chinese	Reference	-
Non-Chinese	0.11 (-1.22, 1.43)	0.876
**Religion**		
With religion	Reference	-
Without religion	0.30 (-1.20, 1.80)	0.692
**Marital status**		
Married	Reference	-
Never married	-0.56 (-2.55, 1.42)	0.579
Divorced/Separated/Widowed	-1.87 (-3.74, 0.01)	0.051
**Highest educational level**		
University and above	Reference	-
Below University	-0.78 (-2.00, 0.44)	0.211
**Children**		
Without children	Reference	-
With children	0.69 (-1.14, 2.52)	0.463
**Living with**		
Not living alone	Reference	-
Living alone	-0.03 (-2.21, 2.15)	0.982
**Diabetes mellitus**		
No	Reference	-
Yes	0.03 (-1.66, 1.71)	0.974
**Hypertension**		
No	Reference	-
Yes	1.23 (-0.34, 2.82)	0.123
**Hyperlipidaemia**		
No	Reference	-
Yes	0.10 (-1.47, 1.67)	0.901
**Cancer**		
No	Reference	-
Yes	4.70 (1.12, 8.26)	**0.01**
**Stroke**		
No	Reference	-
Yes	-0.48 (-3.72, 2.76)	0.772
**Personal serious illness requiring hospitalisation**		
No		
Yes	Reference	-
	-0.62 (-2.17, 0.92)	0.43
**Family members with serious illness requiring hospitalisation**		
No		
Yes	Reference	-
	1.90 (0.77, 3.03)	**0.001**

*β represents adjusted linear regression coefficients. Positive coefficients indicate higher perceived benefit scores compared with the reference category.

Table 8 shows the associations between demographic and health-related variables and burden attitudes towards ACP. Overall, most sociodemographic and health-related factors were not significantly associated with attitudes towards the burdens of ACP. Age, gender, ethnicity, religion, marital status, having children, living arrangements, diabetes, hyperlipidaemia, cancer, stroke, personal serious illness, and having family members with serious illness requiring hospitalisation showed no significant associations. In contrast, individuals with **below-university education (p** = **0.023) and hypertension (p** = **0.008)** reported significantly greater perceived burdens of ACP.

**Table 8:** Multivariable linear regression of factors associated with perceived ACP burden score.

Demographics	B (95% CI)	p-value
**Age**		
21-40	Reference	-
41-60	-0.09 (-1.33, 1.15)	0.887
61 and above	-1.08 (-2.56, 0.39)	0.15
**Gender**		
Male	Reference	-
Female	0.61 (-0.33, 1.56)	0.204
**Ethnicity**		
Chinese	Reference	-
Non-Chinese	0.67 (-0.42, 1.75)	0.227
**Religion**		
With religion	Reference	-
Without religion	0.14 (-1.09, 1.37)	0.82
**Marital status**		
Married	Reference	-
Never married	-1.46 (-3.08, 0.17)	0.079
Divorced/Separated/Widowed	0.26 (-1.27, 1.80)	0.737
**Highest educational level**		
University and above	Reference	-
Below University	1.16 (0.16, 2.16)	**0.023**
**Children**		
Without children	Reference	-
With children	-0.36 (-1.85, 1.14)	0.643
**Living with**		
Not living alone	Reference	-
Living alone	-0.40 (-2.19, 1.38)	0.659
**Diabetes mellitus**		
No	Reference	-
Yes	-0.81 (-2.19, 0.57)	0.249
**Hypertension**		
No	Reference	-
Yes	1.74 (0.45, 3.03)	**0.008**
**Hyperlipidaemia**		
No	Reference	-
Yes	0.04 (-1.25, 1.32)	0.954
**Cancer**		
No	Reference	-
Yes	-2.50 (-5.42, 0.43)	0.095
**Stroke**		
No	Reference	-
Yes	-2.08 (-4.73, 0.57)	0.125
**Personal serious illness requiring hospitalisation**		
No		
Yes	Reference	-
	0.80 (-0.47, 2.06)	0.216
**Family members with serious illness requiring hospitalisation**		
No		
Yes	Reference	-
	-0.32 (-1.25, 0.61)	0.5

*β represents adjusted linear regression coefficients. Positive coefficients indicate higher perceived burden scores compared with the reference category.

## Discussion

Our study examined attitudes toward ACP among patients attending a primary care clinic in Singapore and identified factors associated with perceived benefits and burdens of ACP. Overall, participants demonstrated a **positive attitude toward** ACP, perceiving it as beneficial and minimally burdensome. This finding contrasts with the study by Cheng et al., in which respondents reported lower perceived benefits and greater perceived burdens related to ACP discussions [38]. The more favourable attitudes observed in our study may reflect differences in healthcare context, demographic composition, and increasing public awareness of ACP in Singapore in recent years. National initiatives promoting legacy planning and advance care discussions have also been introduced to improve public awareness and engagement with ACP [36].

Despite the generally positive attitudes observed towards ACP, the factors assessed in this study were only weakly endorsed as barriers overall. Fear of death was the highest-ranked factor, but the absolute level of endorsement remained low, with a mean score of 1.83 out of 5 and only one-quarter of participants agreeing that they were terrified of death. Notably, nearly three-quarters of respondents were either neutral or disagreed with this statement. These findings suggest that fear of death is not a substantial barrier for most primary care patients in our sample, although it remains the most commonly endorsed concern relative to the other factors examined. This observation differs somewhat from the prevailing narrative that death anxiety is a dominant obstacle to ACP engagement in Asian populations. Interestingly, our findings also challenge the assumption that older adults are the group most fearful of death. Instead, younger adults demonstrated substantially higher death anxiety, highlighting the importance of targeting ACP awareness initiatives toward younger and healthier individuals who may not yet perceive ACP as personally relevant. While cultural taboos surrounding death and dying have been widely reported in Asian societies, our findings indicate that many Singaporean primary care patients may be more receptive to discussing ACP than previously assumed. This could reflect increasing public awareness of ACP, greater exposure to healthcare planning initiatives, and evolving societal attitudes towards discussions about serious illness and end-of-life care. Nevertheless, the fact that approximately one in four respondents still reported being fearful of death suggests that culturally sensitive communication remains important when introducing ACP discussions. This finding is consistent with recent Asian studies reporting that death anxiety, cultural taboos surrounding discussions of death, family-centred decision-making, and emotional readiness for end-of-life conversations remain important influences on ACP engagement despite increasing public awareness [5,25,28–30].

Several demographic and experiential factors were significantly associated with **greater perceived benefits of** ACP. In particular, participants with a history of cancer and those who had family members with serious illnesses requiring hospitalisation demonstrated significantly more positive attitudes toward ACP. Exposure to serious illness may increase awareness of the complexities surrounding medical decision-making and the importance of documenting care preferences in advance. Previous studies have similarly shown that personal experiences with serious illness or caregiving responsibilities can increase individuals’ willingness to engage in ACP discussions and planning [17,39]. Such experiences may make the potential benefits of ACP more tangible, including improved communication with family members and greater clarity regarding future healthcare decisions.

Conversely, certain groups perceived **greater burdens associated with** ACP **discussions**, particularly participants with lower educational attainment and those with hypertension. Lower education levels may be associated with reduced health literacy or limited familiarity with ACP concepts, potentially making discussions about future medical decisions appear complex or emotionally challenging [40]. These findings are consistent with prior research demonstrating that individuals with higher educational attainment tend to have greater engagement in ACP and higher rates of advance directive completion [17]. Addressing these disparities will require targeted educational efforts and simplified communication strategies to ensure that ACP information is accessible to individuals with varying levels of health literacy.

Taken together, these findings suggest that although ACP is generally viewed positively within Singapore’s primary care population, **psychological barriers and sociodemographic differences continue to influence engagement**. Integrating ACP discussions into routine primary care consultations and developing culturally sensitive educational initiatives may help address these barriers and promote broader adoption of ACP within the community [25].

### Strengths and limitations

This study demonstrates several notable strengths. It achieved a high response rate of 84.9%, reducing the likelihood of non-response bias and providing a robust sample for analysis. The questionnaire underwent rigorous validation, including forward and backward translation into English, Chinese, and Malay, expert review, and assessment of reliability and internal consistency. Conducting the study within a primary care setting also allowed the inclusion of participants from a broad range of age groups, ethnicities, educational backgrounds, and health statuses, providing valuable insights into ACP attitudes among adults attending public primary care services in Singapore.

However, several limitations should be considered when interpreting the findings. First, the study was conducted at a single public polyclinic and therefore may not fully represent the broader Singapore population. Although the ethnic distribution of participants closely mirrored Singapore's national demographic profile, the sample may differ from the general population with respect to socioeconomic characteristics, healthcare utilisation patterns, educational attainment, and health status. Individuals who attend public primary care clinics may have greater healthcare needs, more frequent contact with healthcare professionals, and different attitudes towards healthcare planning compared with those who seek care predominantly in private primary care settings or who do not access healthcare services regularly. Consequently,

these findings cannot be assumed to represent the views of the entire Singapore population and should be interpreted primarily within the context of public primary care attendees.

Second, the convenience sampling approach may have introduced selection bias, as participation was voluntary and individuals who were more interested in healthcare-related topics may have been more likely to participate. Third, the cross-sectional design precludes causal inference and does not allow assessment of how attitudes towards ACP may evolve over time or in response to changes in health status, personal experiences, or national ACP initiatives. Finally, attitudes were self-reported and therefore subject to recall and social desirability biases, with participants potentially providing responses perceived to be more socially acceptable rather than reflecting their actual beliefs or intentions.

### Actionable strategies

Our findings suggest several practical strategies for improving ACP uptake in primary care. First, ACP discussions could be integrated into routine chronic disease reviews, Healthier SG enrolment consultations, and annual health assessments, allowing ACP to be introduced as part of proactive healthcare planning rather than end-of-life care alone. Second, targeted educational materials should be developed for individuals with lower educational attainment, who were found to perceive greater burdens associated with ACP. These materials should use plain language, visual aids, and culturally appropriate examples to improve understanding and reduce misconceptions. Third,

healthcare professionals involved in longitudinal patient care, including family physicians, nurses, care managers, care coordinators, medical social workers, and allied health professionals, should receive structured communication training to improve confidence in introducing ACP discussions and addressing concerns related to death and serious illness.

Fourth, individuals with personal or family experiences of serious illness may represent an important target group for opportunistic ACP conversations, as these participants demonstrated significantly greater appreciation of ACP benefits. Finally, policy-level approaches may complement patient- and clinician-focused interventions. South Korea's experience demonstrates how supportive legislation can facilitate ACP uptake through the establishment of a formal legal framework, standardized documentation processes, and a national registry for advance directives and life-sustaining treatment plans. While Singapore already possesses a mature ACP programme, further strengthening interoperability across care settings, enhancing accessibility of ACP records within electronic medical systems, and continuing nationwide public education campaigns may help normalize ACP discussions and improve completion rates. Such measures may allow patient preferences to be more readily documented, retrieved, and honoured across healthcare institutions.

### Follow-up study

Building upon the findings of our study, a valuable follow-up study would involve a mixed-methods longitudinal intervention trial across diverse Singaporean healthcare settings – including public and private clinics in multiple regions to evaluate culturally-tailored ACP strategies over 12–24 months. This would compare standard ACP education with an innovative approach reframing discussions around “planning for living well” instead of “end-of-life discussion” using materials adapted to address specific concerns of Singapore’s major ethnic groups. The trial would integrate qualitative interviews with patients and providers to examine relational dynamics in ACP conversations, while tracking changes in attitudes and completion rates. By addressing current limitations in generalizability, causality, and cross-sectional design, this approach could generate robust evidence for implementing culturally sensitive ACP strategies, potentially increasing adoption rates across Singapore’s multi-ethnic population through healthcare system-specific recommendations.

## Conclusion

Patients attending primary care in Singapore generally viewed ACP positively, perceiving high benefits and low burdens associated with ACP discussions. Although fear of death was the most frequently endorsed barrier, overall endorsement was low and decreased significantly with age and exposure to illness-related experiences. These findings suggest that many primary care patients may already be receptive to ACP conversations. Efforts to improve ACP uptake should therefore focus on creating opportunities for engagement rather than solely addressing resistance. Practical strategies include integrating ACP into routine primary care workflows such as chronic disease reviews, Healthier SG enrolment consultations, and annual health assessments; developing culturally sensitive and health literacy-appropriate educational resources; equipping primary care clinicians with communication skills training; and leveraging illness-related encounters as teachable moments for ACP discussions. Together with continued public education and improved accessibility of ACP documentation across healthcare settings, these approaches may help normalise ACP as part of proactive healthcare planning and increase its uptake in the community.
